# Anatomical and Electrophysiological Clustering of Superficial Medial Entorhinal Cortex Interneurons

**DOI:** 10.1523/ENEURO.0263-16.2017

**Published:** 2017-10-16

**Authors:** Joan José Martínez, Bahar Rahsepar, John A. White

**Affiliations:** 1 Department of Bioengineering, University of Utah, Salt Lake City, UT 84112; 2 Department of Biomedical Engineering, Boston University, Boston, MA 02215

**Keywords:** cluster analysis, entorhinal, excitability, interneuron

## Abstract

Local GABAergic interneurons regulate the activity of spatially-modulated principal cells in the medial entorhinal cortex (MEC), mediating stellate-to-stellate connectivity and possibly enabling grid formation via recurrent inhibitory circuitry. Despite the important role interneurons seem to play in the MEC cortical circuit, the combination of low cell counts and functional diversity has made systematic electrophysiological studies of these neurons difficult. For these reasons, there remains a paucity of knowledge on the electrophysiological profiles of superficial MEC interneuron populations. Taking advantage of glutamic acid decarboxylase 2 (GAD2)-IRES-tdTomato and PV-tdTomato transgenic mice, we targeted GABAergic interneurons for whole-cell patch-clamp recordings and characterized their passive membrane features, basic input/output properties and action potential (AP) shape. These electrophysiologically characterized cells were then anatomically reconstructed, with emphasis on axonal projections and pial depth. K-means clustering of interneuron anatomical and electrophysiological data optimally classified a population of 106 interneurons into four distinct clusters. The first cluster is comprised of layer 2- and 3-projecting, slow-firing interneurons. The second cluster is comprised largely of PV+ fast-firing interneurons that project mainly to layers 2 and 3. The third cluster contains layer 1- and 2-projecting interneurons, and the fourth cluster is made up of layer 1-projecting horizontal interneurons. These results, among others, will provide greater understanding of the electrophysiological characteristics of MEC interneurons, help guide future in vivo studies, and may aid in uncovering the mechanism of grid field formation.

## Significance Statement

Despite the critical role that entorhinal inhibitory interneurons play in computation and grid cell formation, the electrophysiological properties of this inhibitory interneuron population remain largely uncharacterized. This study describes systematically the electrophysiology and anatomy of the inhibitory cells in the medial entorhinal cortex (MEC) and introduces a grouping framework for the population. This framework divides the interneuron population into four clusters, based on differences in their axonal projections and electrophysiological properties. These findings confirm and extend findings from previous anatomic studies and will inform future studies of medial entorhinal interneurons.

## Introduction

By modulating the activity of principal neurons, interneurons play a crucial role in the spatial navigation function of the superficial medial entorhinal cortex (MEC; Garden et al., 2008; Varga et al., 2010; Beed et al., 2013; Couey et al., 2013; Domnisoru et al., 2013; Pastoll et al., 2013; Buetfering et al., 2014; Fuchs et al., 2016). Among other findings, recent studies have demonstrated that MEC GABAergic interneurons mediate stellate-to-stellate cell communication (Couey et al., 2013; Pastoll et al., 2013) and that background inhibition of principal cells is stronger in superficial layers of the MEC than the deeper layers (Woodhall et al., 2005). Grid cell computation work has implemented inhibition-dominated network models to simulate spatial navigation mechanisms (Burak and Fiete, 2009; Pastoll et al., 2013; Thurley et al., 2013). Nevertheless, there is uncertainty as to whether interneurons provide location-dependent input onto grid cells, with Buetfering et al. (2014) providing *in vivo* data that supports at least parvalbumin (PV)+interneurons not exhibiting such spatial variability and computational studies of the MEC (Solanka et al., 2015; Shipston-Sharman et al., 2016) contending that other interneuron groups may be providing such a role .

Despite their importance, electrophysiological data for GABAergic interneurons remain scarce (Gloveli et al., 1997; Wolansky et al., 2007; Fuchs et al., 2016). The characterization of superficial MEC interneurons has been difficult for two reasons: the low proportion of interneurons (∼10%) compared to principal cells (Gatome et al., 2010) and the relative physiologic and anatomic diversity of cortical interneuron populations (Maccaferri and Lacaille, 2003; Whittington and Traub, 2003; Buzsáki et al., 2004; DeFelipe et al., 2013). Previous research suggests that the superficial MEC is anatomically diverse, containing at least seven anatomic categories as defined by soma depth and dendritic morphology (Canto et al., 2008). The anatomic differences in the interneuron population are likely to coincide with different roles within the local cortical circuit (Kepecs and Fishell, 2014). Despite the anatomic categorization of MEC interneurons, the combination of low cell counts and functional diversity has made systematic electrophysiological studies difficult with only a few such studies having been attempted (Gonzalez-Sulser et al., 2014; Fuchs et al., 2016; Ferrante et al., 2017). Limited data are available on the firing pattern of basket cells and chandelier cells, both of which have generally lumped together using their common molecular identifier PV (Wouterlood et al., 1995), but these data do not include passive membrane features, basic input/output measures or action potential (AP) characterization. Furthermore, the electrophysiological properties of remaining cell types in the superficial MEC have remained largely unknown (Gloveli et al., 1997; Wolansky et al., 2007).

This study takes advantage of recent developments in transgenic techniques that specifically label GABAergic interneurons to systematically characterize the superficial MEC interneuron population both electrophysiologically and anatomically. Acute brain slices were harvested from glutamic acid decarboxylase 2 (GAD2)+ and PV+ labeled transgenic mice and whole cell patch clamp techniques were used to measure a variety of electrophysiological features. *Post hoc* anatomic reconstruction was then conducted using fluorescence staining and two-photon imaging to couple each interneuron’s electrophysiological profile with its MEC localization and axonal tree distribution. We find that superficial MEC interneurons can be grouped into four separate groups that have distinct anatomic and electrophysiological profiles. These categories include deep-projecting layer 2/3 slow-firing interneurons, layer 2/3 projecting fast-spiking interneurons, layer 1/2-projecting interneurons and layer 1-projecting superficial interneurons. Our results complement recently published data on the relationship between electrophysiological properties and molecular markers (Ferrante et al., 2017). Together, these two papers represent a large step toward a complete characterization of medial entorhinal interneurons.

## Materials and Methods

### Electrophysiology

All electrophysiology experiments were conducted according to protocols approved by the Institutional Animal Care and Use Committees of the university. Brain slices were harvested from 18–35 d old transgenic mice of either sex. Because no effects of age were evident in our dataset, data were not classified by age of the animal. Two transgenic strains were used: cre-dependent GAD2-IRES-tdTomato transgenic mice (Taniguchi et al., 2011; The Jackson Laboratory, strain 010802), which labeled GAD2-expressing cells and thus facilitated targeting of GABAergic cortical interneurons; and PV-tdTomato transgenic mice (Hippenmeyer et al., 2005; The Jackson Laboratory, strain 008069), which labeled all PV-expressing cells and thus facilitated targeting of the specific PV+ genotype in inhibitory interneurons. These mice were anesthetized with isoflurane and decapitated. The brain was then harvested, chilled in sucrose-substituted artificial CSF (ACSF; 185 sucrose, 2.5 KCl, 1.25 NaH_2_PO_4_, 10 MgCl_2_, 25 NaHCO_3_, 12.5 Glucose, 0.5 CaCl_2_), and cut parasagittally into 300-μm-thick slices using a vibrating microtome (Vibratome VT1200; Leica). Slices were incubated for 15 min in ACSF (125 mM NaCl, 2.5 mM KCl, 1.25 mM NaH_2_PO_4_, 10 mM MgCl_2_, 25 mM NaHCO_3_, 25 mM glucose, and 2 mM CaCl_2_) at 37°C, and then allowed to recover for at least 30 min at room temperature. For recordings, slices were transferred to a heated (32–34°C) slice chamber (Warner Instruments) that is mounted on an upright microscope stage (Olympus BX53; Olympus) and perfused with 95%/5% O_2_/CO_2_ ACSF. GAD2+/PV+ neurons were visualized using fluorescence and whole-cell patch clamp clamped using patch pipettes (5–6 MΩ) fabricated from borosilicate glass (1.5 O.D. 1.1 I.D.; Sutter Instruments) and filled with artificial intracellular fluid (120 mM K-gluconate, 5 mM MgCl_2_, 0.2 mM EGTA, 10 mM HEPES, 20 mM KCl, 7 mM di(tris) phosphocreatine, 4 mM Na_2_ATP, and 0.3 mM Tris-GTP) loaded with biocytin (1% by weight) for *post hoc* reconstruction. Presented data were not corrected for the junction potential, which we measured as 11.6 mV. Electrode resistance in the bath was ∼ 5 MΩ. Τhe pipette capacitance was compensated for automatically by the MultiClamp 700B/CV-7 system. The bridge balance was applied to correct for series electrode resistance under whole-cell patch clamp (∼10 MΩ). Cells were patched for at least 30 min to ensure complete biocytin fill. Following electrophysiological trials, brain slices were perfused in 4% paraformaldehyde (PFA) for 16–24 h, then washed in PBS (137 mM NaCl, 2.7 mM KCl, 10 mM Na_2_HPO_4_, and 1.8 mM KH_2_PO_4_) three times for 15 min each and stored in 4° C for later staining.

### *Post hoc* anatomic reconstruction

**Figure 1.**
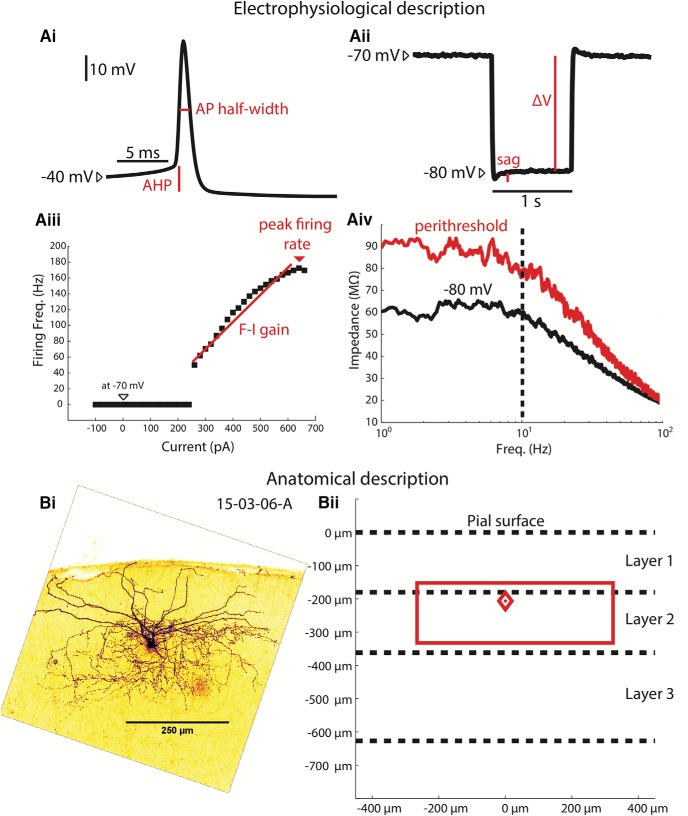
MEC interneuron electrophysiological and anatomic characterization. ***Ai***, Depolarizing current was injected to elicit firing and resulting APs were characterized. AP half-widths are measured at half the height of the AP (using the AP initiation upstroke, or “knee,” as the base). The duration between passing the half-height on the depolarizing phase and passing the half-height on the hyperpolarizing phase is the AP half-width. The AHP is measured as the membrane potential difference between the AP knee and the most hyperpolarized membrane potential immediately following the AP. ***Aii***, One-second-long hyperpolarizing pulses are injected to hyperpolarize the cell from −70 mV to approximately −80 mV. The resulting voltage deflection ΔV is divided by the injected current ΔI to calculate the input resistance. The sag ratio was defined as ΔV/(sag+ ΔV). ***Aiii***, The F-I relationship was described injecting progressively increasing current pulses and measuring the resulting firing rate. The slope between the first non-zero F-I trial and the peak firing trial is the F-I gain. The fastest firing rate elicited by the current pulses is the peak firing rate. ***Aiv***, The impedance spectra are measured at both −80 mV and near threshold are used to calculate the impedance change. The impedance between 1 and 10 Hz of the perithreshold spectrum is divided by the same impedance band of the rest spectrum. The dashed line indicates the upper band for the impedance change calculation, 10 Hz. ***Bi***, A z-stack projection of a biocytin filled, Alexa Fluor 488-labeled MEC interneuron is used to determine the location of the soma and estimate the extent of the axonal tree. ***Bii***, The neuron in Bi is described using a diamond to indicate the depth of the soma (relative to the pial surface) and a rectangle to describe the laminar and columnar extent of the neuron’s axonal projection, both in red. Dashed line indicate the average depth of layers 1–3. This neuron has a soma at the layer 1/2 border (∼200 µm deep), and its axonal tree extends from ∼170 to 350 µm in depth and is ∼500 µm wide.

Brain slices were incubated for 3 h in a PBS solution containing 3 µg/ml streptavidin Alexa Fluor 488 (Invitrogen) and 2% Triton X-100 (by volume). Slices were then washed in PBS three times for 15 min each and mounted on slides using a Mowiol mounting medium. At least 24 h after mounting, slides were imaged using a two-photon microscope (Ultima Intravital, Bruker), with excitation wavelength set to 810 nm and a 520-nm low-pass filter. Alexa Fluor 488-filled cells were localized in the brain slice and a z-stack of 585 × 585 µm raster-scanned images was acquired, covering the entire range of the soma and neuronal processes (usually 100–200 µm). This z-stack was projected onto a single composite image and endowed with a dark-cell, light-background look-up table to aid axonal visualization (Fig. 1). to describe the anatomic features of each neuron, soma depth was measured and the extent of the axonal tree was described with a rectangular approximation using the z-projected image (Fig. 1).

### Electrophysiological protocols and data analysis

We used established methods to characterize the electrophysiological features of patched cells. All electrophysiological protocols were conducted in current clamp and were performed within 30 min of breaking into the cell to begin the whole cell patch clamp recording.

#### Input resistance, time constant, sag ratio, and resting membrane potential

A bias current was applied in current clamp to hold the cell to −70 mV. Five 1-s negative current pulses (with a 2-s rest time) were injected to hyperpolarize the cell to approximately −80 mV (between −20 and −50 pA, depending on input resistance). The resulting steady-state hyperpolarization from −70 mV was divided by the applied current to calculate the input resistance. To determine the membrane time constant, a single-order exponential was fit to the membrane response (from start to peak polarization) to the hyperpolarizing current. The sag ratio was determined by dividing the maximum voltage hyperpolarization (the sag) by the steady state hyperpolarization during the pulse. The resting membrane potential was measured for every cell by placing the cell in current clamp, injecting no current, and measuring the membrane potential.

#### AP half-width, AP rise time, and spike afterhyperpolarizing potential (AHP)

A depolarizing bias current was injected to elicit APs just above firing threshold. The average of 50–100 total APs recorded in a 30–40 s recording was used to describe the AP shape. First, the AP half-width was determined by determining the AP height from the AP upstroke (defined as the point at which the second derivative of the AP with respect to time was at its maximum) to the peak and calculating the duration between passing the half-height on the depolarizing phase and passing the half-height on the hyperpolarizing phase is the AP half-width. The AP rise time was calculated as the time required for the AP to go from 20% of its total height to 80% of its total height. The AHP was measured as the membrane potential difference between the AP upstroke initiation and the most hyperpolarized membrane potential immediately following the AP.

#### Firing threshold

The cell was hyperpolarized to −80 mV and a 50 pA/s current ramp for 10–20 s was applied, depending on the input resistance. The firing threshold was determined as the membrane potential at the upstroke of the first AP in the ramp.

#### Impedance

The cell was hyperpolarized to −80 mV and a 15 s filtered white noise current trace (filtered at 200 Hz and set to 50–100 pA in amplitude to elicit a membrane response ∼5 mV in amplitude) was injected. We used a low pass filtered white noise, as described in Fernandez et al. (2015), where the current signal was constructed in the frequency domain using a frequency amplitude equal to 1/(1+(f/200)), where f is the frequency. The voltage trace and the resulting injected current trace were each converted into the frequency domain (using a fast Fourier transform). The frequency domain of the voltage was divided by the frequency domain of injected current trace and magnitude component of the resulting trace was used as the impedance. This procedure was repeated several times at increasing depolarized membrane potentials until the cell was near its firing threshold. The impedance change was measured by calculating the average impedance between 1 and 10 Hz for the most depolarized trace (labeled “perithreshold”) and dividing it by the average impedance for the −80 mV trace in the same frequency band.

#### Frequency-current (F-I) gain, peak firing rate, minimum firing rate, adaptation ratio, and rheobase

A bias current was applied in current clamp to polarize the cell to −70 mV. A series of one-second current pulses (with a four second rest between pulses) was injected to determine the F-I relationship of the cell. These current pulses ranged from −100 to up to 1500 pA, depending on what current amplitude was required to reach a firing rate plateau, and were introduced in 20 pA increments. The peak firing rate was the fastest firing frequency recorded during the F-I trial. The minimum firing rate was measured as the lowest non-zero firing rate recorded during the trial. This measurement illustrates any large discontinuities in spike frequency associated with the spike threshold transisiton. To determine the F-I gain, we determined both the F-I trial point at which the minimum firing rate was achieved and the F-I trial point at which the firing rate asymptote began. A linear regression fit for all the F-I trials between these two points (inclusively) was calculated with the least-squares “polyfit” function in MATLAB for a first order polynomial. The slope of this fit was taken to be the F-I gain. The adaptation ratio was measured as the ratio of the first three interspike intervals to the last three interspike intervals in the median firing F-I step. For example, if an F-I trial had 11 steps in which the interneuron fired at least one spike, then the 6th trial would be used to measure the adaptation ratio. The rheobase was estimated using the F-I trial by detecting the first current pulse to elicit an AP. The magnitude of that current pulse was taken to be the rheobase for that cell.

### Grouping methodology

#### Principal component analysis (PCA)

PCA (Jolliffe, 2002) was used to prevent correlations between the different measured values in the electrophysiological/anatomic dataset. The electrophysiological features used for this analysis were: input resistance, peak firing rate, rising time constant, change in impedance and F-I gain. These electrophysiological measurements were selected as they represent both the passive and active membrane properties of the cell populations, while not including several measurements that could be correlated. The anatomic features used were: soma depth, the most superficial extent of the axonal tree, the deepest extent of the axonal tree, and the axonal width. Because including rheobase and resting membrane potential had minimal effect on the outcome of k-means clustering described below (changing the classification of only 4/106 cells), we did not include these variables. All these features were z-scored (i.e. mean-subtracted and divided by the standard deviation) before the analysis. Each cell was treated as an observation with each feature a variable. The “princomp” function in MATLAB was used to calculate the transformation. We used four principal components to account for 80% of the variance. Increasing this number did not change the k-means classification outcome. Using three, two, or one principal component changed the k-means outcome for 3, 39, and 45 of 106 classified cells. Thus, we conclude that using three to four components is optimal for this dataset, and used four to meet the standard criterion of accounting for 80% of variance.

#### K-means clustering analysis

K-means clustering analysis (MacQueen, 1967) was used on the first four principal components of the above dataset to group cells. The “kmeans” function in MATLAB 2016a was used with squared Euclidean distance as the metric, the kmeans++ seeding algorithm (Arthur and Vassilvitskii, 2007), and 100 iterations for each operation to ensure convergence. Silhouette scores were calculated using the “silhouette” function in MATLAB. The silhouette score is a measure of similarity of a point to points within its own cluster and of dissimilarity of a point to points outside of its own cluster (Rousseeuw, 1987). For a given cell *i*, it is calculated as s(*i*) = (b(*i*)-a(*i*))/maximum[a(*i*),b(*i*)], where a(*i*) is the average distance between cell *i* and all other cells in its cluster and b(*i*) is the shortest distance between cell *i* and any cell not in *i*'s cluster. The range of values ranges from −1 to 1. A higher score (closer to 1) indicates high similarity within cluster and dissimilarity outside of cluster, whereas a lower score (closer to −1) indicates low similarity within cluster and dissimilarity outside of cluster (suggesting the data point was misclassified).

#### Group comparisons

When comparing electrophysiological and anatomic features among different cell groups, reported *p* values were calculated using a the nonparametric Kruskal-Wallis one-way ANOVA test. The *F* value and *p* values for all comparisons are shown in Table 1.

**Table 1.** Statistical tests

Characteristic	*F* value	*p* value
Input resistance (MΩ)	72.93	1.0E-15
Time constant, falling (ms)	75.86	2.4E-16
Time constant, rising (ms)	76.05	2.2E-16
Rebound amplitude (mV)	25.08	1.5E-05
Firing threshold (mV)	12.26	6.5E-03
Resting membrane potential (mV)	37.26	4.1E-08
A.P. rise time (ms)	57.61	1.9E-12
A.P. half-width (ms)	69.29	6.1E-15
Spike AHP (mV)	16.44	9.2E-04
F-I gain (Hz/nA)	42.62	3.0E-09
Peak firing rate (Hz)	74.60	4.4E-16
Lowest firing rate (Hz)	54.33	9.6E-12
Rheobase (pA)	59.90	6.2E-13
Adaptation ratio	22.01	6.5E-05
Change in impedance (%)	14.42	2.4E-03
Sag ratio	33.51	2.5E-07
Soma depth (µm)	42.38	3.3E-09
Axonal tree, superficial (µm)	29.87	1.5E-06
Axonal tree, deep (µm)	29.77	1.5E-06
Axonal tree, width (µm)	25.09	1.5E-05

All characteristics were tested for normality using the Kolmogorov–Smirnov test, and none were found to be normally distributed. The nonparametric Kruskal--Wallis test was used to compare groups, and the *F* value and *p* value for each test is shown.

#### Alternative methods of clustering

In addition to the combined data k-means clustering method used in this study, we explored several different approaches to the clustering problem. First, we used hierarchical clustering to group the interneuron population and compared the results to those arrived at using k-means clustering. The first four principal components of the combined anatomic/electrophysiological dataset (five electrophysiological measures and four anatomic measures) were clustered using the unweighted pair group method with arithmetic mean (UPGWA) for hierarchical clustering (Sokal, 1958), using the least squared Euclidean distance to separate interneurons. As this method sequentially separates the population into different hierarchies, it is possible from one analysis to group interneurons into a few large clusters or several smaller clusters. For the purposes of comparing our results to those of k-means clustering, the cutoff for differentiating clusters was set to 60% of the maximum distance between any two interneurons. That is, all interneurons within a single cluster have to be no further than 60% of the maximum distance measured in this population; interneurons with a greater distance must be in separate clusters.

Second, we explored clustering the interneurons using either only anatomic data or electrophysiological data, as opposed to combining both data types into one analysis. We separated out the four anatomic and five electrophysiological measures and conducted separate analyses. For both sets of data, we z-scored the measurements and conducted PCA, as described previously. For k-means clustering analysis, we set the cluster number to four, to match with the optimal cluster number for the combined analysis. The optimal cluster number was determined by varying the set number of clusters and measuring the mean silhouette score of all the clusters. The number of clusters that yielded that highest mean silhouette score was set to be the optimal cluster number.

To compare different distributions, it was necessary to determine the optimal correspondence between clusters of one distribution to that of the combined k-means clustering distribution used in the study. We established the optimal correspondence by testing every possible permutation for assignment overlap (percentage of interneurons assigned to the same cluster in both distributions), and choosing the permutation of highest overlap.

### Validation of transgenic mouse models

In order to validate the transgenic mice line, immunohistochemistical analysis was performed. Mice were injected with an overdose of pentobarbital sodium/phenytoin sodium (Euthasol, Vibrac Animal Health) and trans-cardially perfused with 0.05 M PBS followed by 4% PFA in 0.05 M PBS. Brains were removed and post fixed in the PFA for 4 h. Brains were transferred to 30% sucrose solution in 0.05 M KPBS solution and incubated over night to be cryoprotected. Each brain was flash froze in OCT and sliced into 40-μm horizontal slices. Slices from GAD2-TdTomato and PV-Tdtomato animals were, respectively, stained with markers for GAD2 (Invitrogen, PA5-22260) and PV (Swant, PV25) followed by Alexa Fluor 488 goat-antirabbit secondary antibody. Imaging was performed using two-photon imaging system (Thorlabs) with a mode-locked Ti:Sapphire laser (Chameleon Ultra II; Coherent) set to wavelengths between 900 and 925 nm using 20× water immersion NA 1.0 (Olympus) objective lens. For the PV slices, z-stacks were taken by imaging at 0.5-μm intervals through the regions interest of the slice. For the GAD2 slices, image was taken at a single plane. Cells were counted by analyzing two nonoverlapping regions of MEC on each slice for each animal.

## Results

### Interneuron characterization

Interneurons of the mouse superficial MEC were systematically patched, electrophysiologically characterized, and then anatomically reconstructed to better understand the local inhibitory components of this brain region. Because interneurons make up a small (<10%) portion of all the MEC (Gatome et al., 2010), transgenic mice labeling GAD2+ and PV+ cells were used to target the neuron subpopulation. For each interneuron, passive properties (like input resistance at rest, time constant, and sag ratio) and active properties (like AP shape and F-I relationships) were measured in the current clamp configuration (Fig. 1*A*), and the neuron was stained *post hoc* with an Alexa Fluor 488 fluorescent marker. The neuron was then reconstructed in a three-dimensional z-stack using a two-photon microscope (Fig. 1*B*). In all, each neuron had sixteen electrophysiological features and four anatomic features recorded (Table 2).

**Table 2.** Electrophysiological and anatomic characteristics for all four interneuron clusters with statistical comparisons

Cluster	1	2	3	4	1,2	1,3	1,4	2,3	2,4	3,4
*n*	30	29	16	31						
Input resistance (MΩ)	220.2	85.7	274.4	155.1						
	12.3	5.1	14.9	6.2	*p* < 0.01	n.s.	*p* < 0.05	*p* < 0.01	*p* < 0.01	*p* < 0.01
Time constant, falling (ms)	13.7	5.1	15.8	8.9						
	0.8	0.2	0.9	0.4	*p* < 0.01	n.s.	*p* < 0.01	*p* < 0.01	*p* < 0.01	*p* < 0.01
Time constant, rising (ms)	14.1	5.2	16.2	9.5						
	0.8	0.2	1.0	0.4	*p* < 0.01	n.s.	*p* < 0.01	*p* < 0.01	*p* < 0.01	*p* < 0.01
Rebound amplitude (mV)	0.75	0.41	1.48	0.62						
	0.08	0.03	0.20	0.10	*p* < 0.05	n.s.	n.s.	*p* < 0.01	n.s.	*p* < 0.01
Firing threshold (mV)	−38.7	−36.7	−40.8	−35.6						
	0.9	1.0	1.1	1.0	n.s.	n.s.	n.s.	*p* < 0.05	n.s.	*p* < 0.05
Resting membrane potential (mV)	−66.3	−71.4	−55.5	−66.8						
	1.0	1.0	2.1	1.1	*p* < 0.01	*p* < 0.01	n.s.	*p* < 0.01	*p* < 0.05	*p* < 0.01
A.P. rise time (ms)	0.273	0.192	0.253	0.292						
	0.006	0.003	0.013	0.009	*p* < 0.01	n.s.	n.s.	*p* < 0.01	*p* < 0.01	n.s.
A.P. half-width (ms)	1.085	0.526	0.832	1.151						
	0.038	0.010	0.067	0.047	*p* < 0.01	n.s.	n.s.	*p* < 0.01	*p* < 0.01	*p* < 0.05
Spike AHP (mV)	16.2	19.9	19.3	20.8						
	1.0	0.5	1.0	0.7	*p* < 0.05	n.s.	*p* < 0.01	n.s.	n.s.	n.s.
F-I gain (Hz/nA)	153.0	281.9	373.9	162.0						
	18.9	15.8	51.4	13.0	*p* < 0.01	*p* < 0.01	n.s.	n.s.	*p* < 0.01	*p* < 0.01
Peak firing rate (Hz)	57.4	279.2	111.5	75.3						
	5.1	8.6	8.4	5.6	*p* < 0.01	*p* < 0.01	n.s.	*p* < 0.01	*p* < 0.01	n.s.
Lowest firing rate (Hz)	10.6	79.2	13.7	13.9						
	2.3	6.0	3.0	2.3	*p* < 0.01	n.s.	n.s.	*p* < 0.01	*p* < 0.01	n.s.
Rheobase (pA)	185.7	380.7	118.8	262.6						
	15.7	22.2	12.3	13.2	*p* < 0.01	n.s.	*p* < 0.05	*p* < 0.01	*p* < 0.05	*p* < 0.01
Adaptation ratio	0.783	0.878	1.335	0.917						
	0.153	0.007	0.730	0.153	*p* < 0.01	n.s.	n.s.	*p* < 0.01	*p* < 0.05	n.s.
Sag ratio	0.936	0.945	0.888	0.936						
	0.006	0.004	0.013	0.008	n.s.	*p* < 0.05	n.s.	*p* < 0.01	n.s.	*p* < 0.01
Change in impedance (%)	64.8	133.1	136.5	51.6						
	10.6	12.7	15.7	9.2	*p* < 0.01	*p* < 0.01	n.s.	n.s.	*p* < 0.01	*p* < 0.01
Soma depth (µm)	327.0	304.9	274.6	191.6						
	14.2	16.1	19.7	8.9	n.s.	n.s.	*p* < 0.01	n.s.	*p* < 0.01	*p* < 0.05
Axonal tree, superficial (µm)	296.4	168.4	107.0	114.0						
	21.5	19.8	33.2	25.2	*p* < 0.01	*p* < 0.01	*p* < 0.01	n.s.	n.s.	n.s.
Axonal tree, deep (µm)	446.4	298.0	318.0	252.9						
	23.0	16.4	35.6	25.7	*p* < 0.01	*p* < 0.05	*p* < 0.01	n.s.	n.s.	n.s.
Axonal tree, width (µm)	361.4	513.0	440.6	472.2						
	21.7	12.8	30.8	20.1	*p* < 0.01	n.s.	*p* < 0.01	n.s.	n.s.	n.s.

The electrophysiological and anatomic characteristics of all four clusters are shown, along with the associated *p* values from a Kruskal--Wallis test (as described in Materials and Methods). Each row shows the average value for a different electrophysiological or anatomic measurement. The standard error associated with that measurement is located below the average value. Each column for the left half of the table shows the measurements for each of the four clusters. On the right half of the table, the *p* value for the Kruskal--Wallis test is shown for matched pairs. For example, column “1,2” shows the *p* values for the test between clusters 1 and 2 for each characteristics; *p* < 0.05 are highlighted in blue. Tests that showed no significance are shown as n.s.

**Figure 2.**
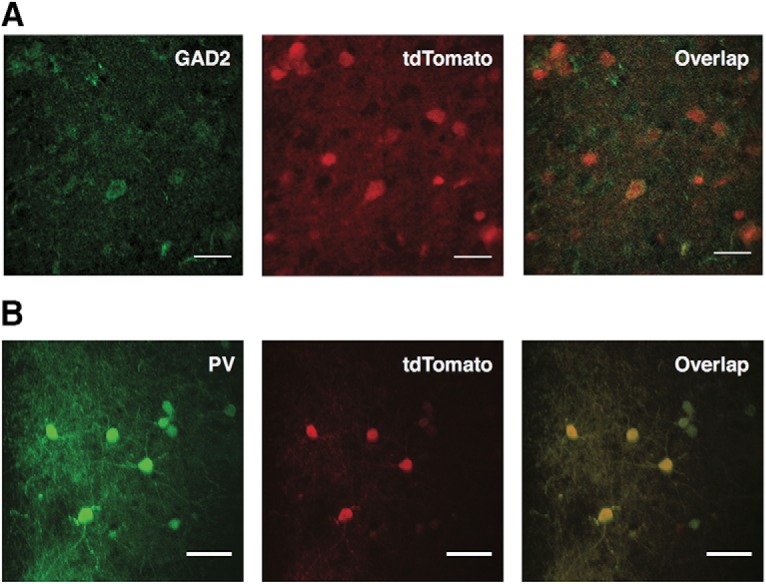
Immunohistochemical validation of transgenic mouse lines. ***A***, Representative image showing overlap of GAD2 labeling with tdTomato cells of transgenic mice. The great majority of the tdTomato-positive cells are labeled for GAD2 as well (91.2% of 74 cells counted; *n* = 2 animals). Scale bar: 25 μm. ***B***, Representative image showing overlap of staining for PV with tdTomato cells. Nearly all tdTomato-positive cells are labeled for PV as well (97% of 74 cells counted; *n* = 2 animals). Scale bar: 15 μm.

**Figure 3.**
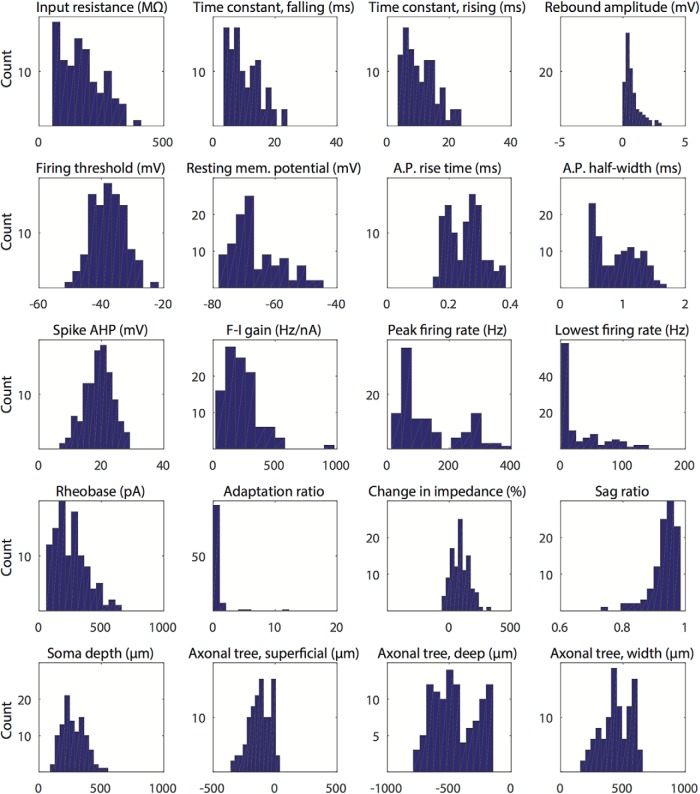
Histogram distributions for electrophysiological and anatomic characteristics. The distributions of the 20 electrophysiological and anatomic characteristics for all 106 superficial MEC interneurons are displayed in histogram form, with each characteristic binned into 12 groups.

The *in vitro* immunohistochemical validation of the transgenic lines showed that the vast majority (91%) of GAD2-tdTomato-positive cells were labeled by GAD2 markers. Similarly, nearly all (97%) PV-tdTomato-positive cells were labeled by PV markers. Both results can be seen in Figure 2. The study yielded a total of 106 interneurons with complete electrophysiological and anatomic profiles. For the 106 cell population, the distribution of each measured characteristic is shown in Figure 3. Of these, 80 cells were acquired using GAD2+ mice and 26 were acquired in PV+ mice. Cells that had incomplete or inadequate electrophysiological trials were not analyzed. Common causes of incomplete electrophysiological characterization included cell death during experiment, incomplete pipette to cell seal, and noise artifacts that corrupted the data acquisition. Cells that exhibited large changes in resting membrane potential at any point during the trials were discarded. Cells that had incomplete anatomic reconstructions, particularly those where the axonal tree was not visible, were also discarded. Common issues with anatomic reconstruction included incomplete anatomic fills and inadequate staining.

### Classifying interneurons into distinct groups

#### K-means clustering

**Figure 4.**
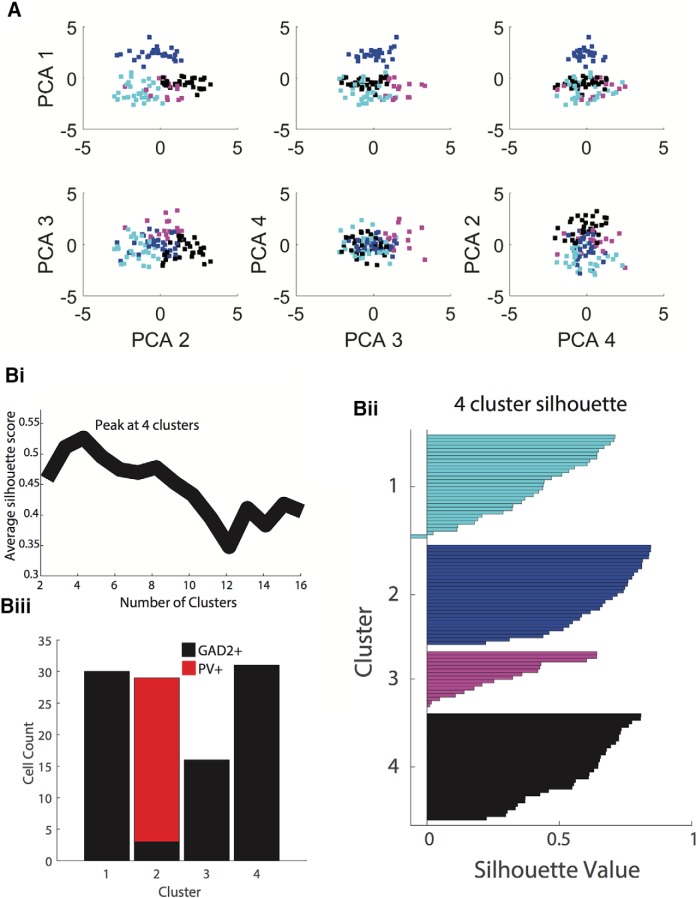
PCA and k-means clustering analysis. ***A***, The first four principal components of combined electrophysiological and anatomic data are plotted in all possible combinations. These four principal component dimensions were used to conduct k-means clustering analysis. Cluster 1 is cyan, cluster 2 is blue, cluster 3 is magenta and cluster 4 is in black. ***Bi***, To determine the optimal number of clusters for k-means clustering, the average silhouette score (measure of distance for within-cluster points compared to outside-of-cluster points) was calculated for k-means clustering analyses using between 2 and 16 clusters. The highest silhouette score was achieved using four clusters, suggesting that this is the optimal cluster number. ***Bii***, The silhouette value (score) for each point is shown in their corresponding cluster. Low or negative silhouette values indicate points that fit poorly within its cluster. ***Biii***, In a four-cluster analysis, PV+ cells were located entirely in cluster 2, with 26 out of 29 cells being PV+. This again suggests that using four clusters for the k-means clustering analysis is optimal.

Given that the large dataset included 106 interneurons each with 16 electrophysiological features and four anatomic features, PCA was used to reduce the variation of the data into fewer dimensions. PCA was conducted using five selected electrophysiological and four anatomic measurements (see Materials and Methods). Since this diverse set of measurements vary greatly in mean and variance, all measurements were z-scored to standardize the PCA variables to a mean of 0 and variance of 1. To reduce the dimensionality of the dataset, subsequent analyses used only the top four ranked principal components, which altogether accounted for80% of the variability in the data. The first four principal components accounted for 29.4%, 24.7%, 16.7%, and 9.2% of the variability, respectively. The next five principal components accounted for 6.7%, 4.7%, 3.7%, 2.7%, and 1.9% of variability, respectively. The relationships among the four principal components have been plotted in Figure 4*A*.

The resulting principal components were then used to group cells into distinct clusters. K-means clustering analysis was performed as described previously. Given that k-means clustering requires the number of clusters as an input, it was necessary to first determine the optimal number of clusters in which to divide the dataset. K-means clustering was thus conducted on a range of cluster number inputs, from only two clusters to up to 16 clusters. For each cluster number input, a silhouette score was calculated for all cells. The silhouette score is a measure of the cluster “fit”: it is high when a data point (in this case a cell) is more similar to data points within its cluster than those outside of its cluster. The average silhouette score for all 106 interneurons in each of the 2–16 cluster k-means analyses was calculated to validate the cluster fit (Fig. 4*B*i). The highest mean silhouette score was achieved when four clusters were assigned to the dataset, suggesting that the analysis is optimal with four clusters.

The resultant silhouette scores are shown in Figure 4*B**ii*. The GAD2-PV cell distribution for these clusters is shown in Figure 4*B**iii*. Cluster 1 has a total of 30 cells, cluster 2 has 29 cells, cluster 3 has 16 cells, and cluster 4 has 31 cells. Notably, the k-means clustering analysis placed all 26 PV+ cells in the dataset into cluster 2. The fact that all PV+ cells were placed in a single cluster and that the cluster itself was almost entirely (26 out of 29, 90%) comprised of verified PV+ cells lends further support to the PCA/k-mean clustering method used in this study. This result also suggests that PV+ cells are a very homogeneous group, representing a relatively small fraction of the GABAergic cells, and anatomically and electrophysiologically distinct from the others.

#### Hierarchical clustering

**Figure 5.**
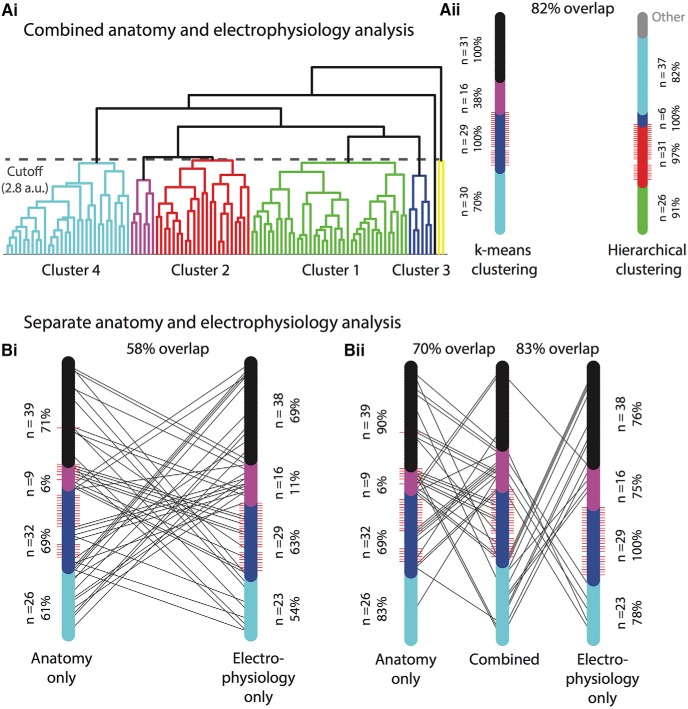
Comparison of different clustering methods: k-means clustering versus hierarchical clustering, combined versus separate anatomic and electrophysiological analysis. ***A***, UPGWA hierarchical clustering using combined anatomic and electrophysiological data yielded similar results to k-means clustering. ***Ai***, UPGWA hierarchical dendrogram separates the 106 interneurons sequentially by the least squared Euclidean distance. Each branching point represents the splitting of a cluster into two clusters, until the clusters are comprised of single neurons. Each end point thus represents a single interneuron. Branch points above the height of 2.8 (a.u.), in this case representing 52% of the maximum distance in the population, are considered to represent distinct clusters. These resulted in eight different clusters. To match up these clusters with those derived from the k-means clustering analysis, all possible permutations were tested. The permutation with maximum overlap, shown per the labels for each cluster, was used for further analysis. ***Aii***, The clustering distribution for hierarchical clustering is shown on the left bar, with each color corresponding to the branch on the dendrogram. The four clusters with less than four interneurons were grouped into the gray “other” category. The clustering distribution for the k-means clustering is shown on the right, in the same color scheme used throughout the rest of the chapter. For each distribution, red whiskers represent the PV+ interneurons. Black lines connect corresponding interneurons that were categorized differently in each distribution, therefore fewer lines indicate greater overlap between clustering methods. The two clustering methods showed 82% overlap, meaning 82% of interneurons were categorized within the same cluster. The number of interneurons in each cluster is noted beside each cluster, along with the percentage of that cluster which was classified into their corresponding cluster in the other clustering method. For example, cluster 1 in the hierarchical clustering method has 26 interneurons, 91% of which were also classified into cluster 1 in the k-means clustering method. ***B***, K-means clustering was used to cluster all 106 interneurons using only one type of data: either anatomic or electrophysiological. ***Bi***, Distribution plots for purely anatomic clustering and purely electrophysiological clustering are shown as in ***Aii***. Both anatomic and electrophysiological clustering were matched to the combined, four-cluster k-means clustering distribution, as described in Materials and Methods. The overlap between purely anatomic and purely electrophysiological clustering was 58%, indicating that some, but not most, interneurons could be matched to different anatomic and electrophysiological profiles. ***Bii***, The same anatomic and electrophysiological distributions as in ***Bi*** are shown in comparison with the combined distribution in the center. There is a 70% overlap between the combined distribution and the anatomic, whereas there is 83% overlap between the combined and electrophysiological distribution.

We conducted UPGWA hierarchical clustering on the same four-dimensional principal component anatomy/electrophysiology data used for k-means clustering in the study. Hierarchical clustering separated the 106 interneuron population into the dendrogram in Figure 5*A*i, with each end point representing a single interneuron and the branch connections indicating linkages between interneurons. This created various levels (“hierarchies”) into which the population could be grouped. Any separation would be based on the minimum required linkage between interneurons for these interneurons to be grouped into the same cluster. By visual inspection, we tested a range of cutoff distances to yield four clusters of similar sample sizes to the k-means clusters used in the study. We therefore set the cutoff at 60% of the maximum distance between any two interneurons in the population. The resulting seven clusters are colored differently in Figure 5*A*i. to adequately compare these clusters with those in the k-means clustering analysis, we inspected all possible permutations (7! = 5040) for maximum overlap. This produced the corresponding k-means cluster labels for the hierarchical clusters shown in Figure 5*A*i.

The hierarchical clustering analysis had 82% overlap with the k-means clustering analysis, meaning 82% of interneurons were placed in the same cluster in both analyses. This result indicates substantial agreement in the results between both methods. The distributions for each clustering analysis are shown in Figure 5*A**ii*. Cluster 1 had 26 interneurons in hierarchical clustering, 91% of which were categorized into the k-means cluster 1; in turn, there were 30 interneurons in k-means cluster 1, 70% of which were classified into hierarchical cluster 1. The interneurons that were not classified into the same cluster are displayed as black lines leading to the corresponding cluster in Figure 5*A**ii*. Both cluster 2 populations contained all PV+ cells, as shown by the red whiskers indicating PV+ cells in Figure 5*A**ii*. Hierarchical cluster 2 had 31 interneurons, 97% classified into k-means cluster 2; in turn, k-means cluster 2 had 29 interneurons, all of which were classified into hierarchical cluster 2. This shows a high degree of agreement in the predominantly PV+ cluster 2. Cluster 3 in hierarchical clustering had 6 interneurons, all of which were classified in cluster 3 of the k-means analysis. The k-means cluster 3 had 16 interneurons, only 38% of which were classified into cluster 3 in hierarchical clustering (the theoretical maximum given the different group sizes). Hierarchical cluster 4 had 37 interneurons, 82% of which were classified into k-means cluster 4. This cluster had 31 interneurons all of which were classified into hierarchical cluster 4.

#### Separate k-means clustering of anatomic and electrophysiological data

We conducted separate analyses using only either anatomic or electrophysiological data using the same PCA and k-means clustering analysis as the study. We matched the resulting four clusters from each analysis to the combined, four-cluster k-means analysis.

First, we compared the clustering results using either only the anatomy or electrophysiology data (Fig. 1*B*i). These distributions showed only a 58% overlap, suggesting that there is limited predictability for anatomy given knowledge of electrophysiology, and vice versa. 61% of anatomic cluster 1 cells (*n* = 26) matched up with electrophysiological cluster 1 (*n* = 23), which in turn had 54% of its cells matched. Similarly, 69% of anatomic cluster 2 cells (*n* = 32) matched with electrophysiological cluster 2 (*n* = 29), of which 63% matched. Electrophysiological cluster 2 had all of the PV+ cells, which indicates that these cells could be well clustered using only electrophysiological data. Anatomic cluster 2, however, had a smaller fraction of PV+ (21 out of 26, 81%) cells, suggesting that anatomic data were not as clear-cut a differentiator for PV+ cells. Anatomic cluster 3 was small, with only nine cells, only 6% of which matched with electrophysiological cluster 3. This cluster had 16 cells, only 11% of which matched with anatomic cluster 3. Overall cluster 3 showed almost no correlation between its anatomy and its electrophysiology. Cluster 4 showed more correct matches between the anatomic (*n* = 39, 71%) and the electrophysiological (*n* = 38, 69%) distributions.

We then inspected the similarity of each separate analysis to the combined distribution used in the study, as shown in Figure 5*B**ii*. The anatomic distribution had 70% overlap with the combined distribution, whereas the electrophysiological cluster had 83% overlap with combined distribution. Anatomic clusters were matched with their corresponding combined clusters in proportions of 83%, 69%, 6%, and 90%, respectively. Electrophysiological clusters were matched with their corresponding combined clusters in proportions of 78%, 100%, 75%, and 76%, respectively.

### Description of the four interneuron groups

#### Assigned groups express distinct electrophysiological/anatomic profiles

The clustering method classified the dataset into four interneuron groups with distinct combinations of anatomic and electrophysiological profiles. Table 2 shows the average soma depth and the average axonal tree extent for each cluster. The average soma depth varies slightly among clusters, with most of the variance being within cortical layer 2. The average axonal extent, however, is distinct among clusters, with each cluster projecting to a different range of cortical layers. Table 2 also shows the electrophysiological characteristics of all the clusters. Overall, no two clusters show similar electrophysiological profiles; although for certain features two clusters may have distributions with substantial overlap, the combination of electrophysiological features for each cluster is unique.

#### Cluster 1: layer 2/3-projecting slow-firing interneurons

**Figure 6.**
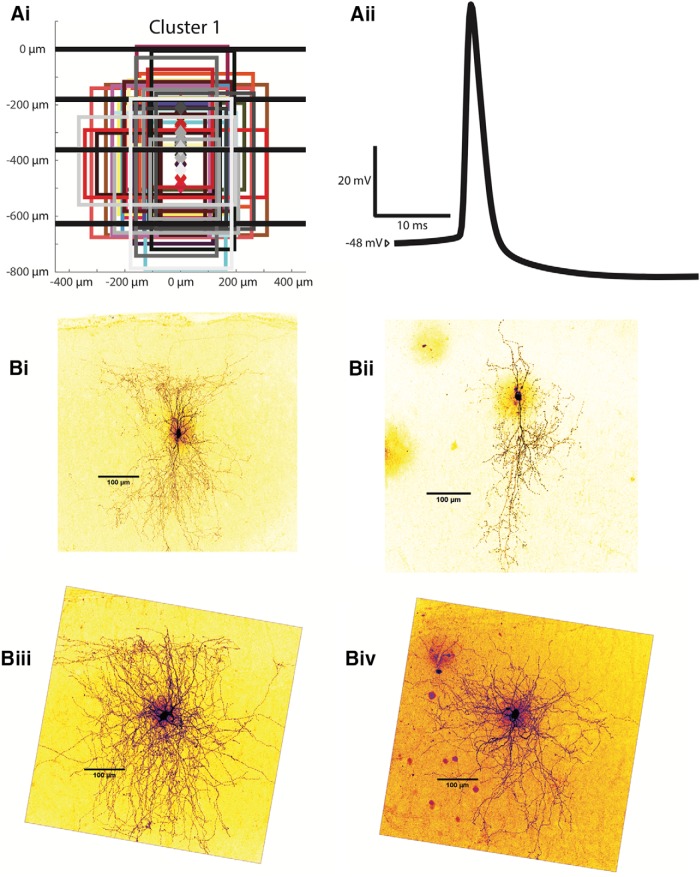
Examples of cluster 1 interneurons. ***Ai***, Anatomic characteristics for all cells in cluster 1 are shown in the same abstract form as in Figure 1*Bii*. ***Aii***, An example of an averaged AP from a characteristic cluster 2 interneuron. ***Bi-Biv***, Z-stack projections of cluster 1 Alexa Fluor 488-labeled MEC interneurons are shown as examples.

Cluster 1 interneurons (*n* = 30) have somas throughout layers 2 and 3, with an average depth of 327.0 ± 14.2 µm. Their axonal projection reach layers 2 and 3, and their average axonal extent is the deepest of all clusters at 296.4 ± 21.5 µm (*p* < 0.01) for its most superficial extent and 446.4 ± 23.0 µm (*p* < 0.05) for its deepest extent. The average axonal width is narrower (*p* < 0.01) than cluster 2 and 4 at 361.4 ± 21.7 µm. Several examples of interneurons belonging to this cluster are shown in Figure 6, with all somas and axonal trees shown in Figure 6*A*i and four reconstructions in Figure 6*B**i-Biv*.

**Figure 7.**
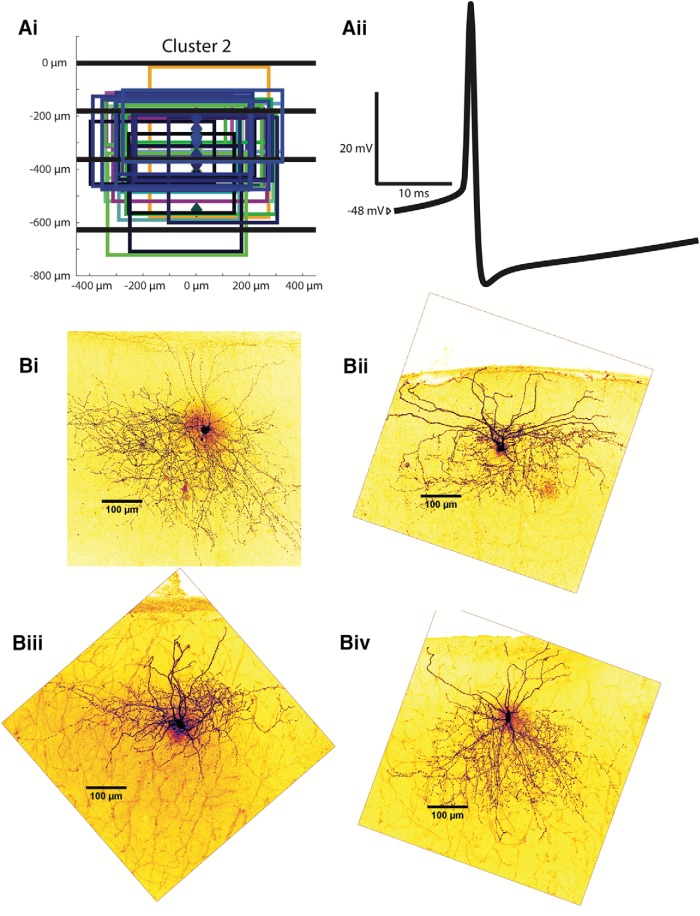
Examples of cluster 2 interneurons. ***Ai***, Anatomic characteristics for all cells in cluster 2 are shown in the same abstract form as in Figure 3.1*Bii*. ***Aii***, An example of an averaged AP from a characteristic cluster 2 interneuron. ***Bi-Biv***, Z-stack projections of cluster 2 Alexa Fluor 488-labeled MEC interneurons are shown as examples.

Cluster 1 is similar to cluster 4 electrophysiologically, with a slow firing rate and flat F-I relationship. The interneurons in this cluster had both the lowest F-I gain at 153.0 ± 18.9 Hz/nA (*p* < 0.01 with respect to clusters 2 and 3) and peak firing frequency at 57.4 ± 5.1 Hz (*p* < 0.01 with respect to clusters 2 and 3). The average input resistance of cluster 1 interneurons is 220.2 ± 12.3 MΩ, the second highest and significantly different from clusters 2 and 4 (*p* < 0.05). The mean falling (rising) time constant is 13.7 ± 0.8 ms (14.1 ± 0.8 ms), significantly longer than in cluster 2 (*p* < 0.01) and shorter than in cluster 3 (*p* < 0.01) but not significantly different from neurons in cluster 4. The resting membrane potential for cluster 1 interneurons averaged at −66.3 ± 1.0 mVm more depolarized than cluster 2 (*p* < 0.01) but more hyperpolarized than cluster 3 (*p* < 0.01). The mean rheobase was 185.7 ± 15.7 pA, lower than both clusters 2 and 4 (*p* < 0.05). The average lowest firing rate was 10.6 ± 2.3 Hz. The mean firing threshold is −38.7 ± 0.9 mV, not significantly different to any other cluster. The AP rise time average for cluster 1 interneurons is 0.273 ± 0.006 ms, and its AP half-width is 1.085 ± 0.034 ms, significantly greater than in cluster 2 (*p* < 0.01). A sample spike shape from a cluster 1 interneuron is shown in Figure 7*A**ii*. Cluster 1 neurons have the shallowest spike AHP of all clusters except cluster 3 at 16.2 ± 1.0 mV (*p* < 0.01). They have a smaller change in impedance between -80 mV and the perithreshold region than clusters 2 and 3 (*p* < 0.01), with an average percentage change of 64.8 ± 10.6 (%). The average sag ratio for the interneurons of this cluster is 0.936 ± 0.006. The adaptation ratio averages 0.783 ± 0.153. In all, the interneurons in cluster 1 are characterized by axonal projections throughout layers 2 and 3 and the slowest firing rates of all interneuron clusters except cluster 4. The interneurons are differentiated from cluster 4 mainly by their greater input resistances and time constants.

#### Cluster 2: layer 2/3-projecting fast-firing interneurons

Cluster 2 is the only cluster containing PV+ interneurons, which comprise 26 out of the 29 cells in this group. Somas of cells in cluster 2 are located throughout layers 2 and 3, with an average depth of 304.9 ± 16.1 µm. Like those from cluster 1, cluster 2 axonal projections are located mainly throughout layers 2 and 3. The most superficial/deepest axonal projections are on average 168.4 ± 19.8 µm and 298.0 ± 16.4 µm, respectively, which places them significantly more superficial than those from cluster 1 cells (*p* < 0.01). Average axonal width is 513.0 ± 12.8 µm. Figure 7*A*i shows all axonal trees for this cluster, and in Figure 7*B**i-Biv*, there are several examples of two-photon reconstructions.

Cluster 2 interneurons are predominantly fast-spiking, in agreement with previous findings on PV+ cells (Jones and Bühl, 1993). The interneurons in this cluster have a very high peak firing rate of 279.2 ± 8.6 Hz, significantly higher than cells from all other clusters (*p* < 0.01) and 250% greater than the next highest spiking firing cluster (cluster 3 at 111.5 ± 8.4 Hz). The average input resistance of cluster 2 interneurons is 85.7 ± 5.1 MΩ, by far the lowest of all clusters (*p* < 0.01). The resting membrane potential for cluster 2 interneurons averaged at −71.4 ± 1.0 mV, more hyperpolarized than all other clusters (*p* < 0.05). The mean rheobase was 380.7 ± 22.2 pA, lower than all other clusters (*p* < 0.05). The lowest firing frequency is significantly higher than all other clusters at 79.1 ± 8.6 Hz (*p* < 0.01). The falling (rising) time constant is also smaller than all other clusters (*p* < 0.01) at 5.1 ± 0.2 ms (5.2 ± 0.2 ms); as are the AP half-width (0.526 ± 0.010 ms, *p* < 0.01) and the AP rise time (0.192 ± 0.003, *p* < 0.01). A sample spike shape from a cluster 2 interneuron is shown in Figure 7*A**ii*. Its 19.9 ± 0.5 mV spike AHP is similar to those from clusters 3 and 4, but significantly greater than AHPs from cluster 1 (*p* < 0.05). Despite its fast firing rate, the F-I gain is only the second highest of the four clusters at 281.9 ± 15.8 Hz/nA, greater than in cluster 1 and 4 (*p* < 0.01 for all comparisons). The percentage change in impedance exhibited in cluster 2 neurons is 133.1 ± 12.7 (%), similar to results from cluster 3 but greater than those in clusters 1 and 4 (*p* < 0.01). The average sag ratio is 0.945 + 0.004, and the adaptation ratio is 0.878 ± 0.007, significantly larger than in cluster 1 (*p* < 0.01) and less than clusters 3 (*p* < 0.01) and 4 (*p* < 0.05). The key features of the interneurons of cluster 2 are expression of PV, axonal projections throughout layers 2 and 3, the most hyperpolarized resting membrane potential, and the fastest firing rate and lowest input resistance of all interneuron clusters.

#### Cluster 3: layer 1/2-projecting interneurons

**Figure 8.**
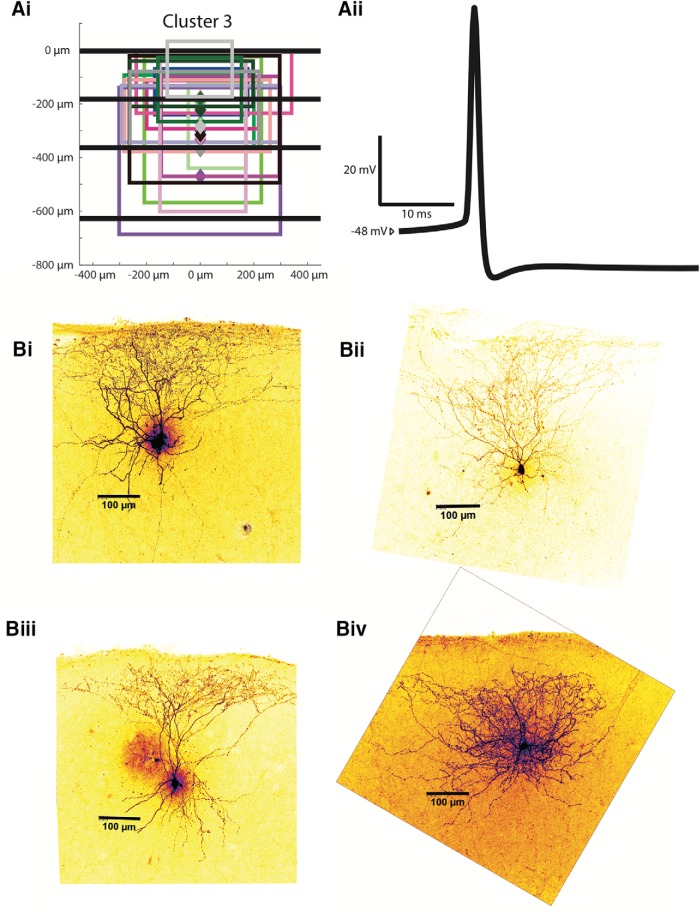
Examples of cluster 3 interneurons. ***Ai***, Anatomic characteristics for all cells in cluster 3 are shown in the same abstract form as in Figure 3.1*Bii*. ***Aii***, An example of an averaged AP from a characteristic cluster 3 interneuron. ***Bi-Biv***, Z-stack projections of cluster 3 Alexa Fluor 488-labeled MEC interneurons are shown as examples.

Cluster 3 interneurons (*n* = 16), like the first two clusters, have somas throughout layers 2 and 3. The average soma depth in cluster 3 is 274.6 ± 19.7 µm. Cluster 3 axonal projections extend mainly through layers 1 and 2. The average axonal extent of cluster 3 interneurons is similar to that from cluster 2 interneurons at 107.0 ± 33.2 µm in its superficial extent and 318.0 ± 35.6 µm in its deep extent (more superficial than in cluster 1, *p* < 0.05). Its anatomic profile is not significantly different from cluster 2 in any respect. Its axonal tree average is 440.6 ± 30.8 µm. Cluster 3 is the smallest cluster, having only 16 cells. All the axonal trees for this cluster are shown in Figure 8*A*i, and several two-photon reconstruction examples are shown in Figure 8*B**i-Biv*.

Cluster 3 interneurons have the highest input resistance (274.4 ± 14.9 MΩ) of any cluster (*p* < 0.01) except cluster 1. Cluster 3 interneurons also have the steepest F-I gain of all clusters except cluster 2 at 373.9 ± 51.4 Hz/nA (*p* < 0.01). The average falling (rising) time constant is 15.8 ± 0.9 ms (16.2 ± 1.0 ms), greater than clusters 1 and 2 (*p* < 0.01). The resting membrane potential for cluster 3 interneurons averaged at −55.5 ± 2.1 mV, more depolarized than all other clusters (*p* < 0.01). The mean rheobase was 118.8 ± 12.3 pA, significantly lower than clusters 2 and 3 (*p* < 0.01). The firing threshold is −40.8 ± 1.1 mV, more hyperpolarized than clusters 2 and 4 (*p* < 0.05). The AP half-width (0.832 ± 0.067 ms) is significantlygreater than cluster 2 (*p* < 0.01) but less than cluster 1 and 4 (*p* < 0.05). The AP rise time is 0.253 ± 0.013 ms, and the spike AHP 19.3 ± 1.0 mV. A sample spike shape from a cluster 3 interneuron is shown in Figure 8*A**ii*. The peak firing rate is 111.5 ± 8.4 Hz, higher than cluster 1 but less than cluster 2 (*p* < 0.01). The lowest firing rate is 13.6 ± 2.7 Hz. The change in impedance for cluster 3 is 136.5 ± 15.7 (%). Cluster 3 has the lowest sag ratio at 0.888 ± 0.013 (*p* < 0.05), the only sag ratio to be significantly different compared to other clusters. The average adaptation ratio was 1.335 ± 0.730, significantly greater than cluster 2 (*p* < 0.05). Cluster 3 interneurons are defined by their axonal projections restricted mainly to layer 1 and 2, as well as having the most depolarized resting membrane potential and a relatively high input resistance and F-I gain.

#### Cluster 4: layer 1-projecting interneurons

**Figure 9.**
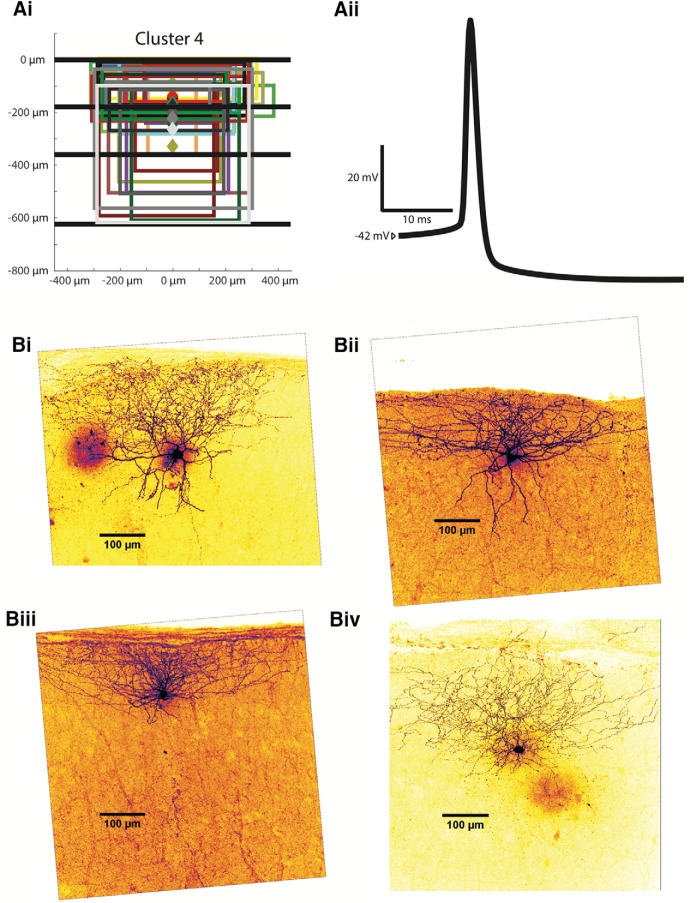
Examples of cluster 4 interneurons. ***Ai***, Anatomic characteristics for all cells in cluster 4 are shown in the same abstract form as in Figure 3.1*Bii*. ***Aii***, An example of an averaged AP from a characteristic cluster 4 interneuron. ***Bi-Biv***, Z-stack projections of cluster 4 Alexa Fluor 488-labeled MEC interneurons are shown as examples.

Cluster 4 interneurons (*n* = 31) have the most superficial somas of any cluster (*p* < 0.01). At an average soma depth of 191.6 ± 8.9 µm, cluster 4 interneuron somas are located throughout layers 1 and 2. This cluster’s axonal projections are mainly limited to layer 1, with its average deepest axonal extent being the most superficial of than cluster 1 (252.9 ± 25.7 µm, *p* < 0.01). The average most superficial axonal extent is 114.0 ± 25.2 µm and the axonal width is 472.2 ± 20.1 µm. The entire population of somas and axonal extents is shown in Figure 9*A*i, and several examples of two-photon reconstructions for cluster 4 interneurons are shown in Figure 9*B**i-Biv*.

Most of the electrophysiological features for these layer 1-projecting interneurons do not lie at either extreme among the clusters. Input resistance is 155.1 ± 6.2 MΩ, greater than in cluster 2 (*p* < 0.01) but less than in clusters 1 (*p* < 0.05) and 3 (*p* < 0.001). The resting membrane potential for cluster 4 interneurons averaged at −66.8 ± 1.1 mV, between clusters 2 and 3 (*p* < 0.05). The mean rheobase was 262.6 ± 13.2 pA, greater than clusters 1 and 3 (*p* < 0.05) but less than cluster 2 (*p* < 0.05). The average F-I gain is 162.0 ± 13.0 Hz/nA, less than clusters 2 and 3 (*p* < 0.01), Average peak firing rate is 75.3 ± 5.6 Hz, less than in cluster 2 (*p* < 0.01). The average lowest firing frequency is 13.9 ± 2.3 Hz, similar to that of cluster 1 and 3. The cluster 4 average falling (rising) time constant is 8.9 ± 0.4 ms (9.5 ± 0.4 ms), and firing threshold is −35.6 ± 1.0 mV. The AP rise time is (0.292 ± 0.009 ms). The AP half-width is 1.151 ± 0.047 ms, greater than those of clusters 2 (*p* < 0.01) and 3 (*p* < 0.05). A sample spike shape from a cluster 4 interneuron is shown in Figure 9*A**ii*. The spike AHP is 20.8 ± 0.7 mV. The percentage change in impedance is 51.6 ± 9.2 (%), lower than clusters 2 and 3 (*p* < 0.01). Finally, the sag ratio for cluster 4 is 0.936 ± 0.008, and the adaptation ratio is 0.917 ± 0.153. Overall, cluster 4 interneurons are characterized by their superficial somas and axonal projections that are relatively limited to layer 1, with electrophysiological features at neither extreme among the interneuron clusters.

## Discussion

**Figure 10.**
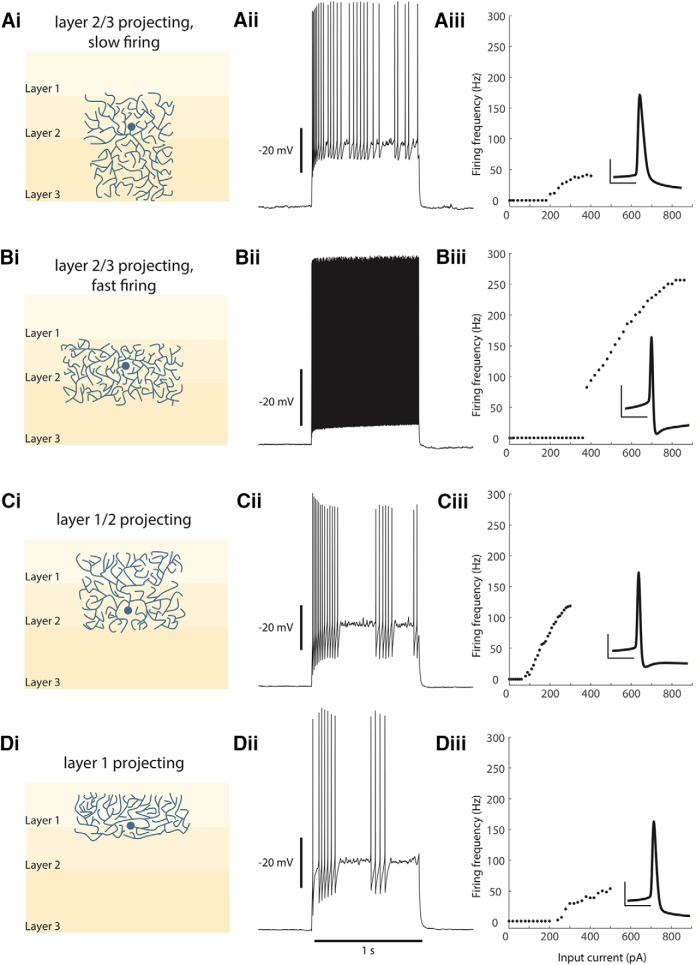
Interneuron groups of the superficial MEC. ***A***, Layer 2/3 projecting, slow firing interneurons. ***Ai***, Representations of the average soma depth and axonal projections are shown. ***Aii*,** A 1-s spike train of a representative cell. ***Aiii***, A typical F-I curve for this population, with the inset showing an average AP (vertical scale bar is 20 mV and horizontal scale bar is 10 ms). ***B***, Layer 2/3 projecting, fast firing interneurons. ***Bi-Biii***, Same as ***A***. ***C***, Layer 1/2 projecting interneurons. ***Ci-Ciii***, Same as ***A***. ***D***, Layer 2 projecting interneurons. ***Di-Diii***, Same as ***A***.

Having systematically characterized 106 interneurons in the superficial MEC, we have found that this interneuron population is best classified into four distinct groups, based on their anatomic and electrophysiological characteristics. In anatomic classifiers, the laminar extent of axonal projection and the somatic depth of interneurons were emphasized. For electrophysiological classification, input resistance, peak firing rate, rising time constant, change in impedance and F-I gain were used. The resulting interneuron groups are layer 2/3-projecting, slow-firing neurons; layer 2/3-projecting, fast-firing neurons (mainly PV+); layer 1/2-projecting interneurons; and layer 1-projecting interneurons. The anatomic and electrophysiological characteristics of each of the interneuron groups are described in Table 2 and summarized in Figure 10.

### Clustering anatomic and electrophysiological data

The clustering method for interneuron data used PCA to reduce the nine-dimensional parameter space into four orthogonal dimensions (principal components) with maximized variance (Jolliffe, 2002). Multidimensional clustering was then performed on the first four principal components and silhouette scores were used to determine optimal cluster number. This method is similar to previous approaches to neuronal classification (Cauli et al., 2000; Krimer, 2005; Dumitriu et al., 2007; Helm et al., 2013). This study is the first, to our knowledge, to combine both anatomic and electrophysiological characteristics in the analysis, as opposed to using only electrophysiological data (Krimer, 2005; Helm et al., 2013) or conducting clustering analyses for different types of data separately and evaluating correlations (Cauli et al., 2000; Dumitriu et al., 2007). This method is often used to differentiate between cell types within distinct molecular subgroups, such as PV+ or somatostatin (SOM)+ interneurons (Halabisky, 2006; Ma, 2006; McGarry, 2010). Our dataset included cells from the general GAD2+ population and the specific molecular PV+ subgroup, and the clustering method was capable of differentiating the two populations using only electrophysiological and anatomic characteristics.

Anatomic classification used axonal properties and excluded dendritic properties. Our approach was based on the clustering system for GABAergic interneurons proposed in DeFelipe et al. (2013). This system places greater importance in the location and spread of axonal arborization of GABAergic interneurons for the purpose of classification. Axonal projections form the basis for circuit connectivity and thus basing interneuronal classification on axonal geometry provides a pragmatic solution to describe the consequential anatomic characteristics of interneurons, avoiding the complexities introduced by including dendritic structure.

Two distinct methods of unsupervised clustering were used in the study, k-means clustering and hierarchical clustering. The k-means method produces independent groupings with no explicit relationship between the different clusters. To yield the optimal grouping, the k-means method requires several trials randomizing initialization conditions. The hierarchical method measures the relationship between each cell in the dataset and iteratively groups them into larger and larger groupings. Unlike the k-means clustering method, hierarchical clustering produces interrelated groupings and does not require predetermining the number of groupings in the dataset. In this study, the results of the k-means clustering were compared to those of hierarchical clustering to check whether the limitations of k-means clustering were significantly affecting the grouping outcome. Hierarchical clustering showed substantial similarity with k-means clustering, with an overlap of 82% between both analyses. This result lends support to the k-means clustering method used in the study, as similar results could be obtained using a different clustering method without the above-mentioned limitations. It is important to note that the hierarchical clustering method produces seven clusters, as opposed to four, which meant that six of the 106 interneurons were not matched to corresponding k-means clusters. This set a ceiling of 94% on the possible overlap between the two distributions.

Anatomic and electrophysiological clustering comparisons suggest there is only limited (58%) overlap between the separate anatomic and electrophysiological profiles of the interneuron population. Cluster 1 was in both distributions the largest cluster and showed higher than average amount of overlap. Cluster 2, as the cluster containing many PV+ cells, also showed higher overlap than average. Cluster 3, however, showed close to no overlap. The greater disparity in cluster size in both the anatomic and electrophysiological distributions suggest that these datasets do not conform particularly to the division into four clusters, but rather may be better fit to three clusters. This result itself suggests that while combining the two data types yields four distinct profiles of interneurons, anatomy or electrophysiology alone would not predict the same number of clusters. Electrophysiological clustering grouped all but one PV+ cell into the same cluster; however anatomic clustering had six PV+ cells assigned to other clusters. Electrophysiology, thus, may be a more reliable predictor of PV expression than anatomy. When comparing the separated analyses to the combined analysis distribution, we observed that the anatomic distribution had a 70% overlap with the combined distribution, whereas the electrophysiological distribution had a 83% overlap.

### GAD2+ and PV+ populations

Previous immunostaining work has found that PV+ interneurons make up ∼50% of the GAD+ population in the superficial MEC (Miettinen et al., 1996). However, analysis of the GAD2+ interneurons characterized in this cluster found a very small percentage of interneurons exhibiting characteristic PV+ electrophysiological/anatomic profiles. The clustering analysis yielded only three interneurons out of 96 that were taken to be anatomically and electrophysiologically similar to PV+ interneurons by being placed in cluster 2. This discrepancy may be explained by issues in the transgenic technique used in this study. PV+ cells may also have been preferentially lost during slicing as compared with GAD2+ cells. GAD2+/tdTomato fluorescence in PV+ neurons may have been lower than in neighboring cells, discouraging patching of PV+ cells. Fortunately, the addition of separate PV+ transgenic animals into the study compensated in part for the relative paucity of PV+ in the GAD2+ patched cell population.

### Cluster 1

The interneurons of cluster 1 have somas located throughout layers 2 and 3; their axonal projections reach into layers 2 and 3, with some neurons having axons projecting into the lamina dessicans (layer 4). Previous anatomic studies have identified MEC layer 2/3 interneurons with similar anatomic characteristics as pyramidal-looking interneurons (Kumar and Buckmaster, 2006), multipolar cells (Gloveli et al., 1997), and bipolar cells (Wouterlood et al., 2000). Pyramidal-looking interneurons in the MEC layer 3 described by Kumar and Buckmaster (2006) have axonal projections mostly concentrated around the cell body in layer 3 and projecting superficially in layer 2, a feature present in some cluster 1 cells (Fig. 7*A*). They are described as having high input resistance (382 ± 47 MΩ), whereas the population average for cluster 1 neurons is also high relative to other clusters (220.2 ± 12.3 MΩ). Gloveli et al. (1997) in turn described pyramidal-looking interneurons in MEC layer 3 as having much lower input resistances of 50.6 ± 5 MΩ, although they maintained their previously mentioned layer 2/3 axonal projections. The relatively low input resistances measured by Gloveli et al. (1997) are likely due to their use of sharp electrodes (as opposed to the patch electrodes used in this study), which have been shown to reduce the input resistance in a cell by 20–40% (Li, 2004). Overall, these results suggest that a significant portion of cluster 1 cells are pyramidal-looking interneurons. Multipolar cells are described similarly by Gloveli et al. (1997), with a low input resistance of 36.8 ± 3.3 MΩ. Unlike the pyramidal-looking interneurons, the axonal projections of these interneurons project further into layer 2 and can project onto layer 1, in addition to projecting intralaminarly in layer 3. This cell type contains SOM+ and cholecystokinin (CCK)+ cells (Wouterlood and Pothuizen, 2000), and like the pyramidal-looking interneuron is also likely represented within the cluster 1 population. Finally, MEC layer 3 bipolar cells described by Wouterlood et al. (2000) may be included in the cluster 1 population as the cells having narrower axonal widths that can project deeper into the lamina dessicans. Cluster 1 neurons account for the superficial MEC’s deeper-projecting interneurons that generally have lower firing rates and F-I gains. This differentiates them from the fast-firing layer 2/3-projecting interneurons and suggests that they play different roles in local circuit modulation.

### Cluster 2

The second cluster described in this study is made up almost entirely of PV+ interneurons. The four out of 30 cells that are not verified to be PV+ may indeed be PV+, as the GAD2+ marker also covers the PV+ cell population (Miettinen et al., 1996). In the MEC, the population of PV+ neurons with somas located in layer 2 (as is the case with most cluster 2 neurons) contains basket cells and chandelier cells (Canto et al., 2008). Basket cells in the MEC were first described by Jones and Bühl (1993), who through unaided patching over several years successfully characterized 12 basket cells, both anatomically and electrophysiologically. In the anatomic description, they described cells with axonal projection mostly within layer 2, as we see for cluster 2 neurons. Electrophysiologically, they described the PV+ interneurons as fast-spiking, and cluster 2 neurons are the fastest spiking population in the present corpus. Additionally, the basket-like interneurons had AP half-widths of 0.51 + 0.05 ms, very similar to the AP half-widths of cluster 2 neurons of 0.526 + 0.010 ms. Finally, the cells in cluster 2 were very likely to exhibit type 2 F-I relationships as shown by having a significantly higher lowest firing frequency than all other clusters. The large minimum firing rate discontinuity is often associated with fast-spiking PV+ cells (Mancilla et al., 2007). These cells are likely to make up the bulk of the cluster 2 interneuron population. MEC horizontal chandelier cells, named for their vertically oriented axonal aggregations, have been described having a vertical axonal extent 100–200 µm long (cluster 2 average is ∼120 µm); the horizontal extent is usually 250–350 µm wide (cluster 2 average is 513.0 ± 12.8 µm, although some are narrower than 350 µm; Soriano et al., 1993). By visual inspection, chandelier cells comprise a smaller fraction of the cells in cluster 2 than basket cells.

Clusters 1 and 2 have similar anatomic distributions (axonal projections mainly in layers 2 and 3) and so are mainly distinguished by their temporal dynamics. Cluster 1 cells have lower firing rates, lower F-I gains and larger time constants than cluster 2 cells. What role might these two interneuron populations play in the superficial MEC? First, fast-firing PV+ neurons like those in cluster 2 have been shown previously to mediate stellate-to-stellate cell connectivity (Couey et al., 2013), provide grid cell-driven recurrent inhibition to the local circuit (Buetfering et al., 2014), and drive theta-nested gamma oscillations (Pastoll et al., 2013). Second, cortical circuits throughout the brain receive a large dynamic range of excitatory inputs, input which is then balanced by an increase in inhibitory inputs (Borg-Graham et al., 1998; Monier et al., 2003; Wehr and Zador, 2003). This coordination occurs over a large dynamic range, meaning the inhibitory dynamics of each circuit is capable of matching excitatory input across this same temporal range. The existence of slow-firing (cluster 1) and fast-firing (cluster 2) inhibitory interneurons with axonal projections within the same layers may thus serve to provide enough sensitivity and dynamic range to address the heterogeneous multimodal inputs that the MEC receives, facilitating the spatial navigation functions that have been described in layers 2 and 3. Third, optogenetic stimulation of either the PV+ cell populations (as in cluster 2) and SOM+ populations (as are likely present in cluster 1) have been shown to produce ictal discharges *in vitro* in the superficial MEC (Yekhlef et al., 2015). Kumar and Buckmaster (2006) also showed that rats treated with pilocarpine showed reduced levels of these two cell types, which directly resulted in hyper-excitability of layer 2 stellate cells. Dysfunction of cells within clusters 1 and 2 may thus play an important role in epilepsy.

### Cluster 3

Cluster 3 interneuron somas are mainly located in the layer 2 somas and have axonal projections into layers 1 and 2. Anatomic studies have described MEC and lateral entorhinal cortex cells with similar anatomic characteristics as multiform neurons, with axons similarly projecting into the white matter (layer 1) and intralaminarly in layer 2 (Tahvildari and Alonso, 2005). Electrophysiological characterization of these cells in the LEC by Tahvildari and Alonso (2005) showed cells with similar time constants (15.8 ± 0.9 ms in this study, where they showed 20.7 ± 1.32 ms) and peak firing rates (111.5 ± 8.4 Hz compared to ∼125 ± 30 Hz). The average firing threshold they measured in the LEC was slightly more depolarized (−45.8 ± 0.5 mV) than that measured in this study in the MEC (−40.8 ± 1.1 mV); input resistance was also considerably lower in the LEC (55.7 ± 6.85 MΩ) than in the MEC (274.4 ± 14.9 MΩ). However, their study used sharp electrodes which introduce leak conductances to the cell membrane (∼6 MΩ vs 80–120 MΩ). The cells of cluster 3 may therefore be related to the multiform cells electrophysiologically characterized in the LEC and anatomically described in the MEC, although to our knowledge never previously described electrophysiologically. Being the cluster with the smallest sample size and most heterogeneous anatomic distribution, it is difficult to ascertain what role cluster three interneurons may play in the MEC. They have the second fastest peak firing rate and steepest F-I gain to the PV+ cluster 2 cells. Given that cluster 3 and cluster 4 both project into layer 1, the relatively slower firing rate of cluster 4 cells suggest that these two populations play the same fast/slow complementary role that clusters 1 and 2 play in layers 2 and 3, increasing the range of inhibitory responses available to respond to excitatory inputs.

Neurons in both cluster 2 and cluster 3 show an increase in impedance as they approach threshold, averaging approximately at 35% increase from rest to the subthreshold. This phenomenon has been described in Economo et al. (2014), and may be due to a persistent sodium conductance that is activated as the cell is depolarized in the subthreshold regime. The presence of this effect suggests that inputs to cluster 3 and 4 neurons are amplified if they arrive when the membrane potential is near threshold.

### Cluster 4

The fourth cluster describes cells with somas in layer 1 (near the layer 1/2 border) and axonal projections mostly restricted to layer 1 with a horizontal extent on average 472.2 ± 20.1 µm. Neurons with these anatomic characteristics have been described previously as both horizontal cells (Germroth et al., 1989) and multipolar cells (Wouterlood et al., 2000). Horizontal cells have been shown to express CCK in the MEC (Schwerdtfeger et al., 1990), whereas layer 1 multipolar cells in the MEC have been described as calretinin (CR)+ (Wouterlood et al., 2000). Both cells have been described as having at least one axonal projection into the deeper layers of the MEC, a feature that was observed in several examples of the cluster 4 neurons. Although Canto and Witter (2012) electrophysiologically characterized layer 1 horizontal and multipolar MEC neurons, their study was focused on principal cells and discarded interneuron-like cells (with shorter AP half-widths) from their analysis. Therefore, to our knowledge, this is the first characterization of these GABAergic, MEC-layer 1-projecting cells. Layer 1 interneurons have been suggested to play a delayed feedback role in cortical computation (Zhou and Hablitz, 1996). Basically, as excitatory inputs arrive from other brain regions and excite pyramidal cells and stellates cells in layers 2 and 3, interneurons in layer 1 may also be excited (either directly by the excitatory inputs or indirectly via principal cells) and inhibit the dendritic branches of the superficial MEC principal cells. Given the larger extentof their axonal projections, it is possible that input to one of these layer 1 interneurons has an effect over a wide area. These may mean inhibitory input onto other layer 1 cells (disinhibition) or inhibitory input onto the dendrites of principal cells in other cortical columns. Further work would be required to understand the specific role these layer 1-projecting cluster 4 neurons play in the MEC.

### Previous findings and future directions

This study emphasizes intrinsic electrophysiological properties and axonal projections in the classification of MEC interneurons. Ferrante et al. (2017) took a complementary approach, analyzing MEC interneurons using several molecular identifiers, including SOM, RCan2, 5HTR3a, and VIP. Like our method, the approach of Ferrante et al. (2017) was effective but included misidentifications; their five electrophysiological parameters together predict biomarker identity with 81% accuracy. Some of these misidentifications may arise from cells that share molecular markers but have different axonal projection patterns and, conceivably, different electrophysiological properties.

Our clusters correspond only partially with those of Ferrante and colleagues. The PV+ interneuron groups in both studies (RCan2 in Ferrante et al., 2017; cluster 2 in this study) were characterized by the lowest input resistance, time constant, and AP half-width and highest peak firing rate when compared to all other interneuron groups. Comparisons with other groups are more difficult. Cluster 1 shares several electrophysiological characteristics with their SOM group, displaying an adaptation ratio of ∼0.65; however our AP half-widths and time constants for cluster 1 are more similar to those of their 5HTR3a interneuron groups. Our cluster 3 was more similar to their SOM group, exhibiting similar AP half-width, adaptation ratio, and time constant characteristics. Cluster 4 does not share an electrophysiological profile with any of the molecular groups in Ferrante et al. (2017). None of the clusters in this study showed as low adaptation ratios as the 5HTR2a interneuron groups, which suggests that these interneuron types were either not covered in the GAD2+ or PV+ cells of this study or are distributed across different clusters. Together, our paper and Ferrante et al. (2017) allow one to make strong predictions of molecular identity and axonal projection pattern based on a rather complete electrophysiological profile, but with some ambiguity and a 10–20% possibility of a mistaken classification.

Our findings point toward several avenues for future research. For example, the interplay between the slow-firing and fast-firing interneuron populations of layers 2 and 3 is a promising target for understanding the grid cell mechanism. Selective optogenetic manipulation of PV+ and SOM+ populations *in vivo* may help explain how the superficial MEC responds to very heterogeneous inputs and generates grid fields. Anatomic work on the layer 1-projecting cells in this study would explain where this interneuronal population receives inputs (whether mainly from other brain regions or local principal cells) and where its main output targets lie (whether mainly principal cell dendrites or other layer 1 interneurons). Furthermore, spatial variations in interneuronal physiology along the MEC’s dorsoventral axis (DVA) could provide vital clues as to the cortical mechanisms behind spatial navigation. Grid field spacing has been shown to increase along the DVA (Hafting et al., 2005). This decrease is matched by a decrease of PV+ inputs and an increase in non-PV+ inputs onto the MEC principal cells (Beed et al., 2010), so there exists an inhibitory gradient along the DVA. Given the known spatial correlates along the DVA, uncovering differences in interneuron physiology (for any of the interneuron populations) between the dorsal end interneurons characterized in this study and the unstudied ventral end interneuronal population would be of particular value to the field.

## Synthesis

Reviewing Editor: Menno Witter, Norwegian University of Science and Technology Decisions are customarily a result of the Reviewing Editor and the peer reviewers coming together and discussing their recommendations until a consensus is reached. When revisions are invited, a fact-based synthesis statement explaining their decision and outlining what is needed to prepare a revision will be listed below. The following reviewer(s) agreed to reveal their identity: Matthew Nolan, Hugh Robinson.

This manuscript describes an electrophysiological and morphological characterisation and classification of interneurons in superficial layers of the medial entorhinal cortex (MEC). The application of clustering algorithms to principal component coefficients obtained from a multidimensional electrophysiological / anatomical characterization provides the possibility to detect the number and characteristics of differerent classes of interneuron. The experiments appear to have been carefully planned and executed and the paper is mostly well-written and well-illustrated.

The conclusion that there are four main clusters or cell types is interesting although is likely to be far from definitive. The study nevertheless has the potential to be of use to the field by providing a quantitative analysis of the diversity of interneuron properties in the superficial MEC and thus contribute towards understanding the detailed circuitry of MEC.

The likely most valuable aspect of the study is not the proposed grouping of neurons, but the dataset that is described. The significance of the publication would be increased substantially if the data were made freely available at time of publication. At a minimum, this should include the dataset used for clustering. Very likely other studies will produce different groupings and rather than going around in circles discussing what this means, publishing the numbers would facilitate quantitative comparisons.

The reviewers have raised a number of major and minor issues that should be addressed in a revised version of the manuscript.

Major

1. Was the specificity of expression of tdTomato in each mouse line validated by co-labelling with appropriate interneuron markers? This seems like an important control which should be included in the study.

2. Please add a figure with histograms to show the distribution of each of the measured variables prior to clustering. It's at present a major weakness of the study that one can not easily assess whether there is any evidence for multi-peaked distributions in any of the measured variables. Please include membrane potential and rheobase in this figure, even if they are not used for clustering.

3. The ages of mice used span time points at which grid firing fields emerge. It would be useful to comment on whether properties change as a function of age.

4. Methods.

a) “A linear regression fit to all points between the first non-zero frequency-current point and the peak firing rate point was calculated with the least-squares ‘polyfit’ function in MATLAB for a first order polynomial”. This seems problematic. To be meaningful the peak firing rate should be an asymptotic value. However, if it is an asymptotic value then a linear fit doesn't make much sense. Please clarify.

b) Measurement of input resistance: “The resulting steady-state voltage was divided...” - presumably the resulting steady-state hyperpolarization from -70 mV was divided ...? Not the absolute voltage as implied. Likewise sag ratio: maximum hyperpolarization should have been divided by the stead-state hyperpolarization, not the steady-state membrane potential, as stated. The method is clearly illustrated in Fig. 1.

c) Impedance: what kind of 200 Hz filter was applied to the white noise stimulus? Presumably low-pass, and what kind of filter characteristics (e.g. Bessel, Butterworth, Gaussian?)?

Why was a straight line fit to the f-I curve used? F-I curves in neurons almost always show sublinearity if the current is sufficiently high, and this is a general property of Hodgkin-Huxley type models as well, as sodium channel inactivation becomes limiting. Indeed, this is seen in Fig 1Aiii. A fit to the initial slope of the f-I curve would have given a better measure of gain - the measure used depends arbitrarily on the level of current at which stimulation was stopped. (If firing frequency reaches a plateau, and then current is increased further, the “gain” will gradually decrease as the peak firing rate point moves horizontally towards higher current values). The approach used seems unnecessarily inaccurate - it would have been equally easy to fit a linear value to the initial segment of the f-I curve, surely.

d) Kmeans clustering - there are many options in Matlab - distance metric, seeding etc. More details including which version of Matlab, are required. I couldn't find any description of how clusters were initially seeded - randomly? Was it tested how the seeding of clusters affected the analysis?

e) Why use the first four principal components equally in the clustering- surely they have very different weights (coefficients) in the PCA? Why not just use the first principal component, if it comprises contributions from all the parameters? The choice of four principle components seems arbitrary - did the authors investigate the effect on the k-means clustering of using fewer or more principle components? If the conclusion that there optimally four clusters is not robust against variation in the number of principle components used, then it would lose some of its meaning.

f) Does it make any difference to the clustering if resting membrane potential and rheobase are included as features? These often show substantial differences between cell types and can be useful for classification. If the analysis can not be repeated with these parameters then please make sure the text explains why not.

5. Please state whether it make a difference to the clustering if fewer or more principal components are used. Please also state the amount of variance captured by each principle component.

6. The statistical analysis states “using a one-way ANOVA with a Tukey's honest significant difference criterion”. Does this mean that an ANOVA was carried out and then a Tukey's test was used for posthoc between group comparisons?

7. Please state in the methods how power and effect size in table 1 were calculated. I'm puzzled that one can have 100% power. These numbers appear to be based on some kind of posthoc calculation. It would be helpful to know if the experimental group size were determined by power calculations prior to experimentation and what the alpha and effect size used for this calculation was.

8. “In order to optimize the clustering analysis, cells with silhouette scores less than 0.2 (shown in a gray dashed line in Figure 2Bii) were discarded from further analysis”. I don't understand the reasoning behind this. It will make the groups appear more discrete than they really are, but then is this an accurate representation of the dataset?

9. How does the grouping based on clustering compare with one based on a hierarchical set of rules? Are there hierarchical rules that lead to groups similar to those found with clustering?

10 Why were dendritic properties not also used for clustering?

11. Increase in impedance towards threshold is striking and unexpected - resembles the phenomenon reported in Economo et al., 2014, Hippocampus 24:1493. Please comment on this phenomenon.

12. For cluster 3 “The lowest firing rate is 13.6{plus minus}2.7 Hz, suggesting type I excitability.” This seems pretty high, are you sure this is not more characteristic of type II excitability?

13. Top of p. 26 - it is argued that the correspondence between hierarchical and k-means clustering might be less good than it could have been because 19 cells were included in the comparison which were discarded from the main k-means analysis for their poor clustering. Isn't this statement easy enough to test precisely, rather than speculate about?

Minor

In the title the use of “clustering” in the title is ambiguous as a reader may not appreciate that this refers to the method used to group the cells. Perhaps “grouping”?

In the abstract, “enabling’ might more accurately be “possibly enabling”.

p 2. “of the superficial medial entorhinal cortex”. Also cite Fuchs et al 2016 and Bead et al. 2013.

p 2. “mediate stellate- to-stellate cell communication”. Should also acknowledge Pastoll et al. 2013.

p 2. “inhibition-dominated network models to simulate spatial navigation mechanisms”. Should also cite Pastoll et al. 2013.

p 2. “argued against the notion that interneurons provide location dependent input onto grid cells”. But see Solanka et al. 2015 and Shipston-Sharma et al. 2016 for counter-arguments.

p 3. “data for GABAergic interneurons remain scarce”. Should also cite Fuchs et al 2016.

p 3. “has made systematic electrophysiological studies difficult”. This has been attempted in Fuchs et al 2016 and Gonzale-Sulser et al. 2014.

p. 4 : Ferrante et al. 2016 citation is not in References. It clearly must refer to a recent paper from the McCormick laboratory in Cerebral Cortex.

p 5. Please measure the liquid junction potential and report this value.

p 5. What was the series resistance and pipette capacitance? Where bridge balance and capacitance neutralisation applied? Where there any criteria for recording quality?

p 7. Should “breaking the cell” be “breaking into the cell”?

p 7. Was the resting membrane potential also measured?

p 7. “The resulting steady-state voltage was divided by the applied current to calculate the input resistance”. Is this correct? Or should it be “The resting membrane potential was subtracted from the resulting steady-state voltage and the result was divided by the applied current to calculate the input resistance”

p 7. “To determine the membrane time constant, a single-order exponential was fit to the membrane response (from start to steady-state) to the hyperpolarizing current”. Is this accurate for neurons with substantial sag? It might be better for these neurons to fit only to the peak polarisation.

p 7. “from the action potential upstroke to the peak”. Please define this more clearly. E.g. Was a threshold applied to the first / second derivative of the membrane voltage, or was some other criterion used?

p 7. It would improve the study if the rheobase could be measured and reported.

p 8. “The average membrane potential in the first trial where a depolarizing current”. Does the average refer to the mode, the median or the mean? This is an unconventional definition of firing threshold, but modal current value might be justifiable.

p 8. “The voltage trace was divided by the injected current trace and the resulting trace was plotted in the frequency domain”. Shouldn't one convert voltage and current traces to frequency domain, then divide and then analyse amplitude and phase components?

”The average of 50-100 .... were used” should be “The average of 50-100 ... was used”

Firing threshold: (p. 8) “The cell was hyperpolarized...” This sentence is missing a main verb - current ramp for 10-20 seconds ...was applied?

p.8, bottom line. “were injected” should be “was injected” (“A series ...”)

p. 9, line 3: “20 nA increments” - presumably 20 pA.

p9. “were introduced in 20 nA increments”. I hope the increments were pA.

p9. “to emphasize variation”. Or to prevent correlations between measured values biasing the clustering procedure?

p11. “size effects” -> “effect sizes”?

p12. The “optimal cluster number” for k-means analysis is referred to here, but not when the analysis is initially described. Please add an explanation for how the optimal number of clusters was calculated.

p. 16, “distributions for each clustering analysis is” should be “distributions for each clustering analysis are”

p. 18, near bottom: “characteristics of all the cluster” should be “... clusters”

p. 19, “peak firing potential at 45.1...” - frequency rather than potential?

p. 21, “The lowest firing frequency is significantly lower” - should be higher.

p. 25 - “Because our dataset included cells...profiles.” I don't really follow the logic of this sentence - what establishes that the method is particularly effective at differentiating different anatomical/electrophysiological profiles?

p. 29 - Jones and Buhl 1993 characterized only 12 basket cells over several years? Is this correct? Or 12 types of basket cell?

p. 30 “is often been associated” should be “is often associated” or “has often been associated”

p32. “though our study” -> “through their study”?

Figure 3 legend, “For example, for Cluster 1...” should be “For example, Cluster 1 ...”
